# Ecological responses to flow variation inform river dolphin conservation

**DOI:** 10.1038/s41598-020-79532-3

**Published:** 2020-12-18

**Authors:** Shambhu Paudel, John L. Koprowski, Usha Thakuri, Rajesh Sigdel, Ram Chandra Gautam

**Affiliations:** 1 School of Natural Resources and the Environment, University of Arizona, 1064 East Lowell Street, Tucson, AZ 85721 USA; 2 Institute of Forestry, Tribhuvan University, Pokhara, Nepal; 3 Haub School of Environment and Natural Resources, University of Wyoming, 201 Bim Kendall House, 804 E Fremont St, Laramie, WY 82072 USA; 4 Vyas Municipality, Government of Nepal, Tanahun, Nepal

**Keywords:** Biodiversity, Ecological modelling, Ecosystem ecology, Freshwater ecology, Hydrology

## Abstract

Many environmental flow (e-flow) studies and applications have predominantly used state—(i.e., at a single time point) and rate—(i.e., temporal change) based demographic characteristics of species representing lower trophic levels (e.g., fish communities) to build flow-ecology relationships, rather than using a process that incorporates population dynamics. Recent studies have revealed the importance of incorporating data on species traits when building flow-ecology relationships. The effects of flow on keystone megafauna species (i.e., body mass ≥ 30 kg) reverberate through entire food webs; however, the relationships between flow and these species are not well understood, limiting the scope of the relationships used in flow management. Here, we fill this gap by incorporating the habitat selection traits at different flows of a freshwater apex predator, Ganges River dolphin (GRD, *Platanista gangetica gangetica*), which plays a significant role in maintaining the structure, functions and integrity of the aquatic ecosystem. Using temporally and spatially measured GRD habitat selection traits, we quantified flow-ecology responses in the Karnali River of Nepal during the low-flow season when habitat was heavily reduced and water demand was highest. We define ecological responses as suitable habitat templates with enough usable surface area to support GRD fitness by improving reproduction and survival. We measured the available and occupied habitats to develop flow-ecology responses. Variation in flow resulted in substantial differences in the ecological response across time and space, suggesting that aquatic species adjusted in a variety of habitats to support their life histories and maintain viable populations. The limited availability of suitable habitats combined with uninformed water regulations by humans likely places GRDs under severe physiological stress during low-water seasons (i.e., January–April), suggesting that  reduced flows contribute to the process of endangering and extirpating highly sensitive endemic aquatic biodiversity. Our study reveals that ad hoc or experience-based flow management is no longer tenable to maintain the integrity and functionality of aquatic ecosystems. We stress that quantifying the flow-ecology relationships of foundational species, particularly megafauna, in response to flow variation is crucial for monitoring the effects of water alterations and determining the minimum flows needed for maintaining healthy and functional freshwater ecosystems in the Anthropocene.

## Introduction

Functionally intact and biologically complex freshwater ecosystems play a critical role in nature and have long-term benefits to society, especially in the Anthropocene^1^. However, hydrological alteration is threatening freshwater ecosystems and their native biotic inhabitants faster than they can be restored^2^. Such modifications affect ecosystems and their aquatic biota in many ways, including having effects on physical habitat, life history, and lateral and longitudinal connectivity^3^. Preserving a freshwater ecosystem’s natural flow regime, in terms of quantity, quality, and seasonality, is essential to protect native biota and environmental processes. Hence, understanding how variation in natural flow drives ecological processes can provide a scientific basis for protecting and maintaining aquatic ecosystems when establishing the appropriate balance between societal and ecosystem needs. For this reason, flow-ecology relationships are globally recognized as a means by which freshwater ecosystems' integrity and dynamic potential can be protected and maintained^3–6^.

The biodiversity of native aquatic species is better maintained in streams in which flow regimes are the most natural^2^. As earth’s larger rivers are obstructed with approximately > 40,000 large dams^7^, the effects of infrastructure on freshwater native biodiversity have been extreme^8^. Previous broad-scale freshwater research focused on the environmental and social consequences of flow variation^9^, but recent studies have emphasized empirical ecology-flow relationships to ensure the sustainability of aquatic ecosystems^10,11^. Previously, e-flow studies and applications relied heavily on regime-averaged metrics (mean seasonal flow characteristics) to explain changes in ecosystem state variables (abundance of species). However, scientists have suggested greater adoption of ecological responses related to process-based (processes contributing to population size) and species traits (e.g., habitat selection traits to improve species fitness) that are rooted in a strong ecological foundation^11–14^. A recent study described these approaches broadly and stressed the importance of performing repeated measurements of ecological responses over time, focusing on reactions that can be linked directly or indirectly to demographic processes^12^.

Flow-ecology relationships can be developed for local or regional scales. Previous studies have typically developed flow-ecology relationships using lower trophic species, such as small fishes and riparian plants, limiting their scope of application. Such relationships might not indicate the full integrity of ecosystems, because megafauna require diverse habitats and are often sensitive to natural flow regimes across a considerable geographic scale. Past studies have often overlooked the essential ecological roles and functions of freshwater megafauna (i.e., body mass ≥ 30 kg;^15^), which help to develop relationships that are transferrable to regional landscapes. The presence of native freshwater megafauna is associated with high native organism biodiversity, and megafauna share common threats with small freshwater species^15^. For these reasons, megafauna-based strategies could potentially maintain key processes and relationships that are robust and able to persist under the anticipated changes in social and environmental conditions^15,16^.

Further, selecting appropriate ecological response variables is key to understanding flow-ecology relationships. Identification of demographic process(es) that strongly influence population dynamics is integral to defining the nature and applicability of relationships^13^. To this point, flow-ecology relationships have generally been developed using multi-species fish models, with the rate of change in species richness as an ecological response^12^. These models overlooked potential trait-based approaches that support ecosystem integrity by maintaining natural flow variability across temporal scales. Further, globally freshwater megafauna also declined by 88% between 1970 and 2012, which was attributed to habitat loss—the primary response to flow alteration changes in habitats^15^. In this paper, we build flow-ecology relationships, assuming associations between reproduction and habitat use, in which habitats with enough usable area act as a template for the reproductive success of species^17^.

Our flow-ecology relationships assume that the GRD, as a freshwater apex predator, is a foundational umbrella species of South Asian aquatic ecosystems (detail ecology, biology and distribution of GRD is available in^18^). If this key species is reduced or extirpated, trophic pathways will change drastically (i.e., there will be higher order effects), and the total ecosystem productivity will decrease^19^. Here, we examine flow-ecology relationships in the Karnali River system of Nepal, a large tributary of the Ganges River in India, by linking GRD ecological responses to flow variation during the low-water season, when hydro-physical habitat is a limiting factor. As a native to South Asian river systems, GRD is distributed from the mouth of Ganges (main river) in India to the foothills of the Himalaya in Nepal. Because of its long-life span and trophic position, the GRD exhibits heterogeneous traits across multiple habitat scales and in critical life-history stages. As a result, the GRD’s status as a keystone predator indicates that it could be a potential bio-indicator for assessing the health of the aquatic system^20–23^. The GRD uses a variety of aquatic habitats across diverse velocities and depths, comprised of stable hydro-physical habitats and eddy counter-currents that trap nutrients and woody debris, thereby enhancing nutrient deposition and aquatic diversity^24^. In line with this hypothesis, GRD habitat-use traits could influence the demographic processes of other aquatic species, and conserving critical habitats of the GRD could improve species diversity and maintain important resources for the rest of the community^25^.

**Figure 1:**
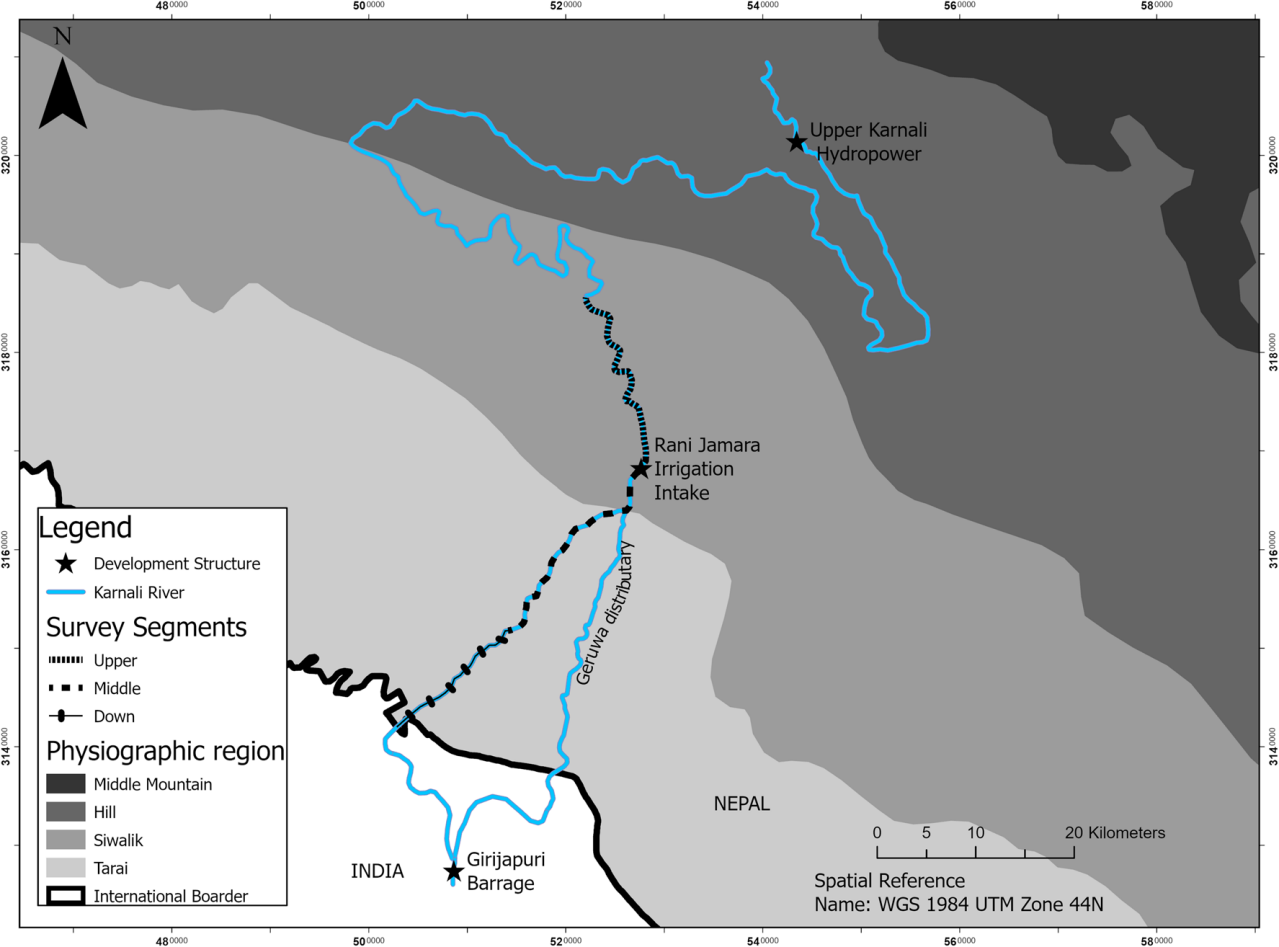
Map of the Karnali River system of Nepal showing locations of Upper Karnali Hydropower, Rani Jamara Kulariya Irrigation Intake and Girirajpuri Dam (in India) that extract the water from the mainstream Karnali River. Survey segments (S1, S2 and S3) represent the historically and presently occupied potential habitat of the Ganges River dolphins. The map was prepared using ArcGIS Pro 2.6 (Esri Inc.; www.pro.arcgis.com), and data (physiographic and river) developed by the Government of Nepal were used to prepare this map.

To develop flow-ecology relationships using GRD habitat selection traits, we first examined GRD habitat selectivity (disproportionately selected habitats) across space and time to develop a habitat suitability curve (HSC) based on data of occupied and available areas. Typically, suitable habitat is estimated based on how often species use a particular habitat across time and space, and it is assumed that such habitats are used by species to maximize their fitness. Developing flow-ecology responses based on apex predator habitat selection traits could be useful for water-resource managers, who are commonly tasked with balancing multiple competing socioeconomic and conservation priorities. Further, by considering the requirements of an apex predator, this research offers generalizable flow-ecology relationships that aid the formulation of flow management guidelines applicable across regional scales that share common species diversity and geomorphic characteristics. This enables the protection of diverse habitats and taxonomic groups by avoiding the risk of crossing ecological thresholds that threaten endemic aquatic biodiversity (Fig. 1).

## Results

### GRD detection based on depth and the velocity of the flow

**Figure 2:**
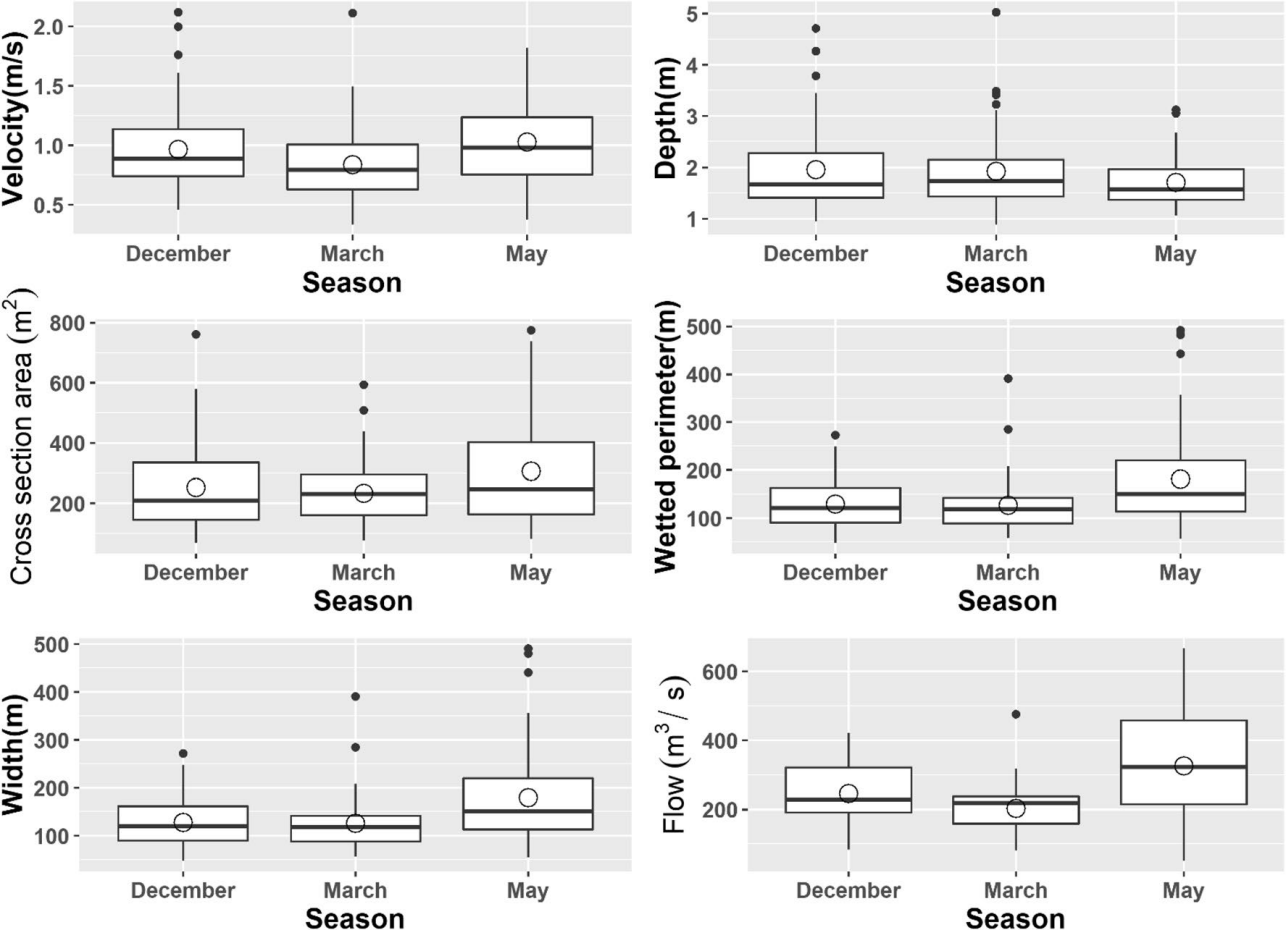
Ganges River dolphins ecological attributes across the dry water season (Dec-early dry, Mar-middle dry and May-late dry; Dry season = December–May; Transition season for both dry and wet respectively = November, June; Wet = July–October) show that individuals are often found in specific hydro-physical habitats, suggesting strong habitat selection. The line’s median value divides the box and means value represented by a hollow circle.

**Figure 3:**
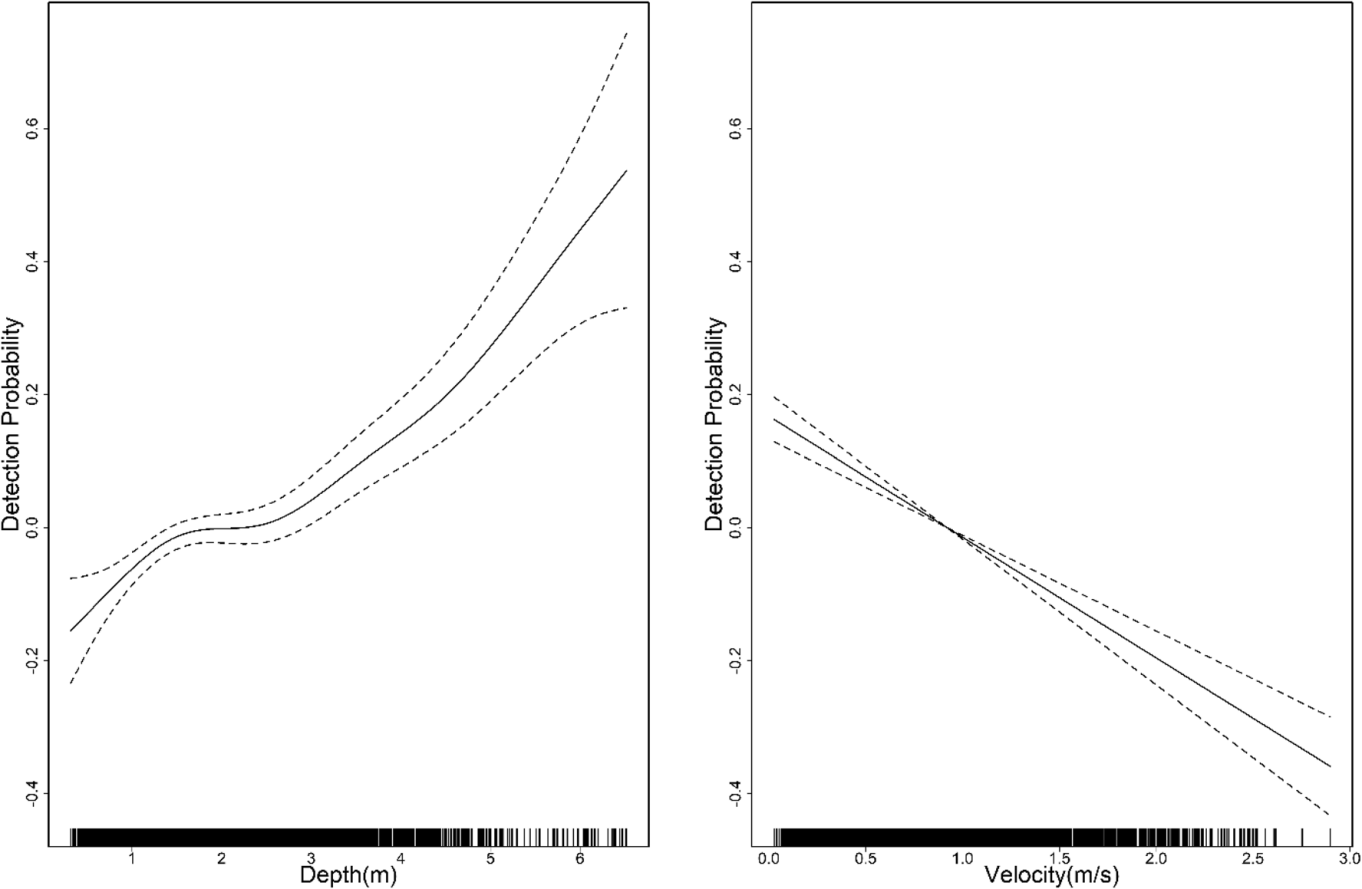
Smoothed curve of the additive effect of depth and velocity to the detection probability of Ganges River dolphins in the General Additive Models, where dotted lines represent 95% CI, and the lower axis represents single observations of depth and velocity, respectively, in the Karnali River of Nepal.

The mean depth of the habitat used was higher in December (mean = 2.119 m, SD = 0.858; preferred range = 3.5–4.2 m, *w* = 1.71, SE (*w*) = 0.13) than in March (mean = 1.953 m, SD = 0.569; preferred range = 6.3–7 m, *w* = 1.9, SE (*w*) = 0.6) or May (mean = 1.620 m, SD = 0.324; preferred range = 4.9–5.6 m, *w* = 9.22, SE (*w*) = 1.62) [Fig. 2]. However, we noticed higher water velocity in the habitat used for May (mean = 0.934 m/s, SD = 0.330; preferred range = 0–0.3 m/s, *w* = 1.67, SE(*w*) = 0.13) than for December (mean = 0.840, SD = 0.228; preferred range = 0.6–0.9 m/s, *w* = 1.9, SE(*w*) = 0.6) or March (mean = 0.788, SD = 0.292; preferred range = 1.8–2.1 m/s, *w* = 3.62, SE(*w*) = 0.47) [Fig. 2]. The model that best predicted the presence of GRD contained the additive effect of depth and velocity [Model 1: depth (AIC = 4665.5, R^2^ = 0.02), Model 2: velocity (AIC = 4637.2, R^2^ = 0.03), Model 3: depth*velocity (AIC = 4560.8, R^2^ = 0.062), and Model 4: depth + velocity (AIC = 4559.1, R^2^ = 0.061)]. We noticed a significant impact of both depth (GLM, β = 1.370, SE = 0.036, Z = 8.671, *p* < 0.001) and velocity (GLM, β =  − 0.454, SE = 0.077, Z =  − 10.114, *p* < 0.001) on the presence of the GRDs. Depths > 2 m had a substantial positive effect on the presence of GRD (Fig. 3). Even though velocity and GRD presence had an inverse relationship, velocities < 1 m/s had a positive effect; velocities greater than 1 m/s had substantial negative effects (Fig. 3).

### The effect of flow fluctuations on hydraulic habitat availability

**Figure 4:**
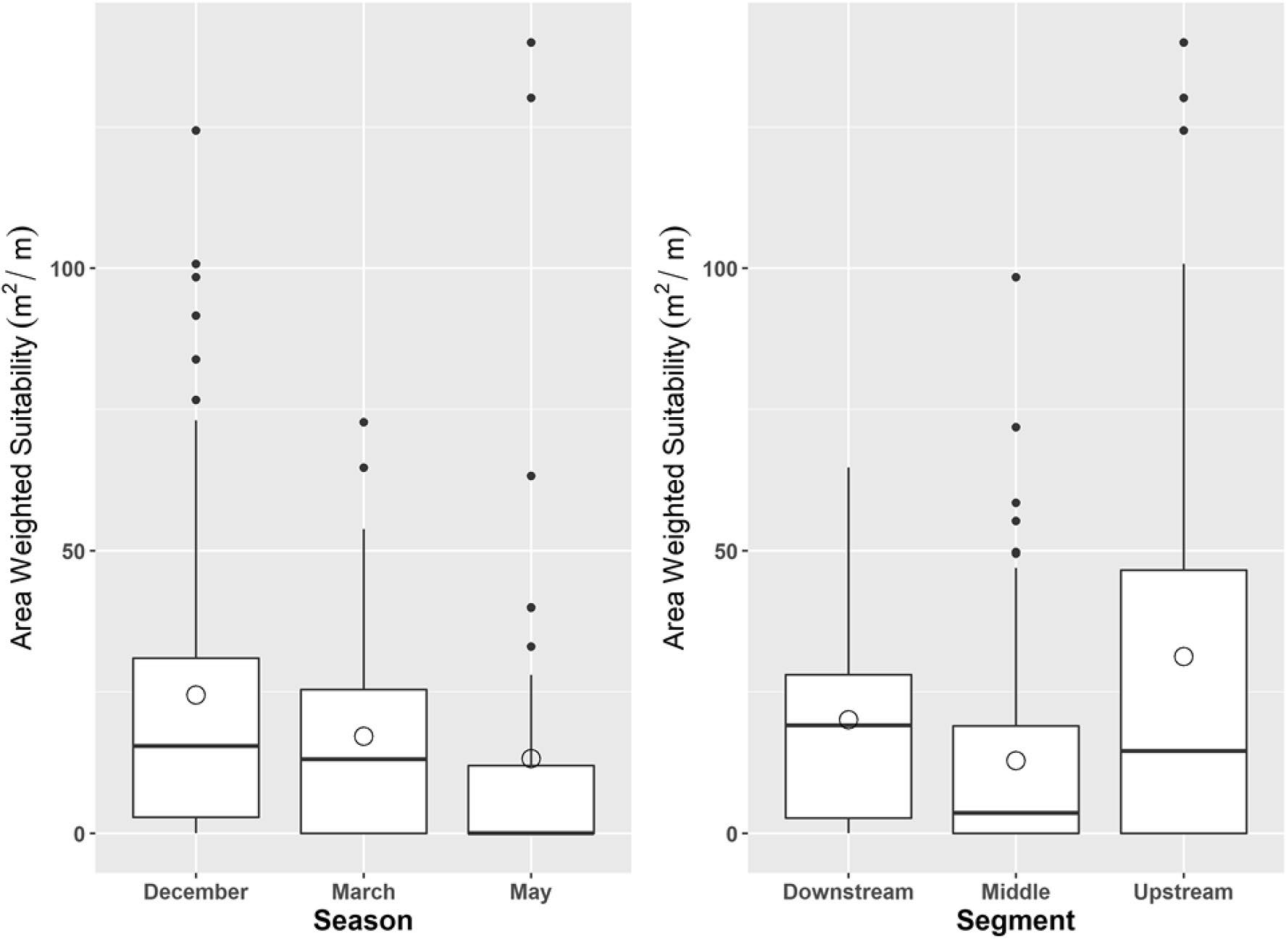
Area weighted suitability (AWS) distribution across dry season (Dec-early dry, Mar-middle dry and May-late dry; Dry season = December–May; Transition season for both dry and wet respectively = November, June; Wet = July–October) and segments (upper, middle and downstream), which shows higher availability of AWS in the confined and unregulated upper segment compared to affected segments from distributaries and human extraction, and seasonally more AWS noticed during the early dry season compared to the middle and late dry season. The line’s median value divides the box and means value represented by a hollow circle.

The mean AWS (suitable physical habitat templates as a function of depth and velocity at particular geomorphologies that offer enough usable surface area to support GRD fitness) recorded in December was higher (24.531 m^2^/m, SD = 29.193, Fig. 4) than that in March (17.220 m^2^/m, SD = 18.759) or May (13.253 m^2^/m, SD = 29.379). The upstream mean AWS (31.330 m^2^/m, SD = 39.401) was higher than that of the midstream (12.884 m^2^/m, SD = 18.755) or downstream segments (20.095 m^2^/m, SD = 18.801, Fig. 4). No significant difference in the average AWS among seasons was observed (ANOVA, F(2, 174) = 2.832, *p* = 0.0616). However, we noticed substantial variation in the average AWS by segment (ANOVA, F(2, 174) = 7.899, *p* < 0.0001, df = 2).

**Figure 5:**
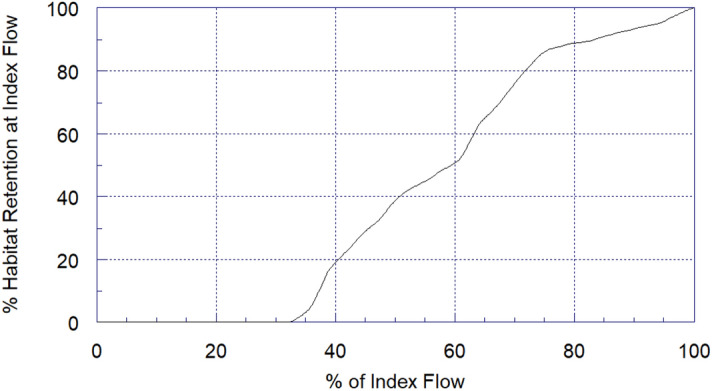
Habitat retention (%) at the different proportion of the base flow. For example, if we maintain 40% of the base flow, at least 20% of the GRDs’ hydro-physical habitats will have remained in the stream.

Fluctuations of 10%, 20%, 40%, and 60% in the current base flow (536.11 m^3^/s) resulted in AWS losses of 2.076 m^2^/m (9% loss of the base flow’s AWS), 3.538 m^2^/m (15% loss of the base flow’s AWS), 6.191 m^2^/m (26% loss of the base flow’s AWS), and 11.998 m^2^/m (50% loss of the base flow’s AWS), respectively (Table 1). For the retention of 80% and 65% of the GRD habitats (or AWS), minimum flows of 383.967 m^3^/s and 348.51 m^3^/s, respectively, are required (Fig. 5, Table 2).

**Table 1:** Area weighted suitability (AWS) loss with flow fluctuations around a base flow of 536.112 cubic meter per second for May.

The proportion of maximum fluctuation	Loss (AWS m^2^⁄m)	% Loss of AWS at base flow
0	0	0
0.1	2.07	8.67
0.2	3.53	14.78
0.3	4.97	20.77
0.4	6.19	25.87
0.5	8.54	35.71
0.6	11.99	50.14
0.7	15.05	62.92
0.8	18.19	76.01
0.9	22.13	92.49
1	23.69	99.02

**Table 2:** Minimum Flows (in cubic meter per second) that retain a % of Area Weighted Suitability at a base flow of 536.112 cubic meter per second.

Retention %	Minimum flows (m^3^/s)
95	503.08
90	446.96
85	398.23
80	383.96
75	372.58
70	361.44
65	348.51
60	337.84
55	330.11
50	317.92
45	294.58
40	270.79
35	258.19
30	244.12
25	229.62
20	216.15
15	205.51
10	198.47

### Quantifying flow-ecology relationships to determine flow regimes

**Figure 6:**
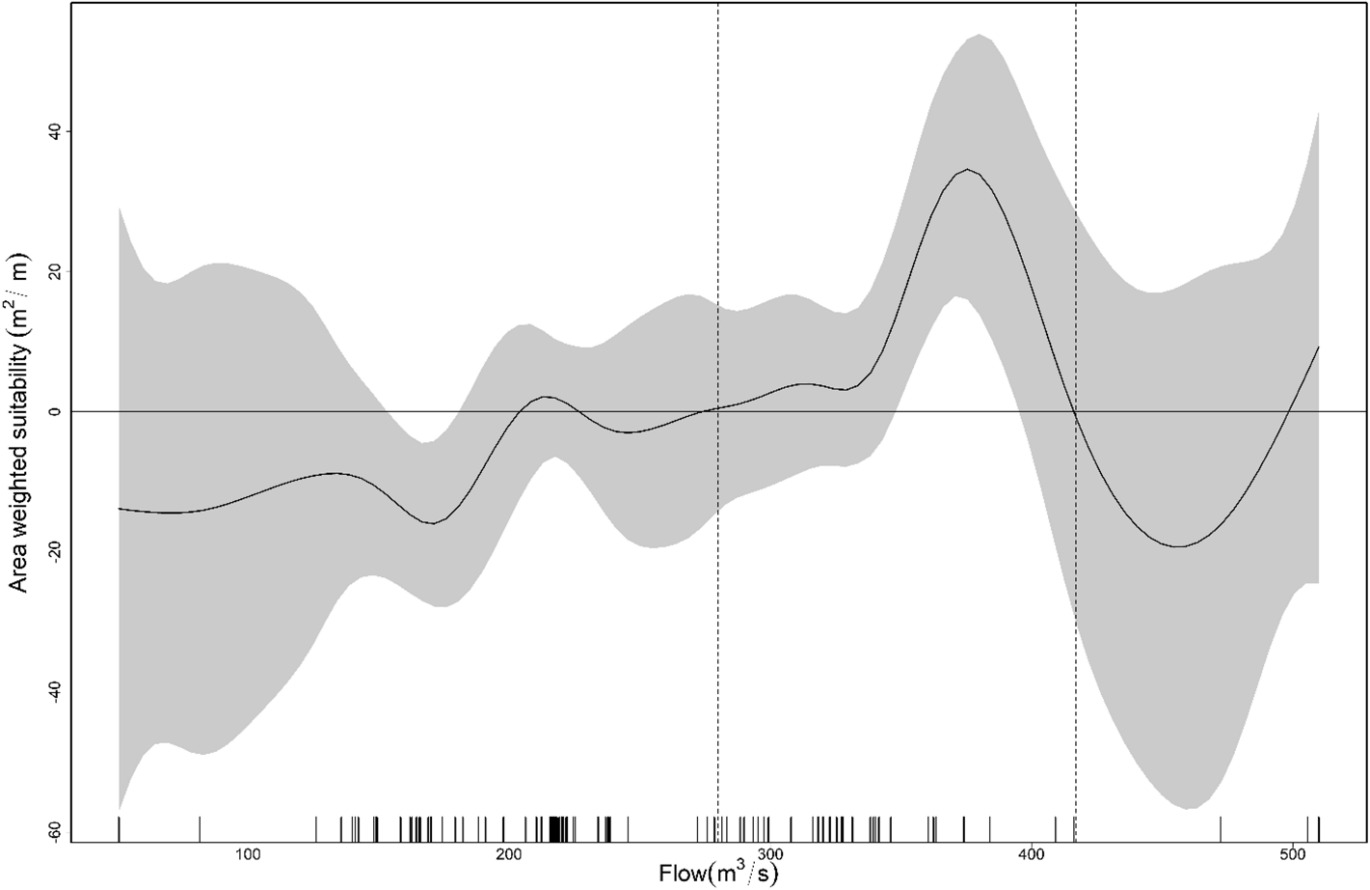
Ecological response (Area Weighted Suitability-AWS) of Ganges River dolphins as a function of flow in a continuous scale, where the smoothed curve line shows the estimated AWS as a function of flow variation, gray color represents the 95% CI level, and marks along the lower axis represent a single observation of the flow level. The two dotted vertical lines identify the limits for the optimum flow range that contribute positively to AWS, whereas the black horizontal line represents the zero effects zone.

**Figure 7:**
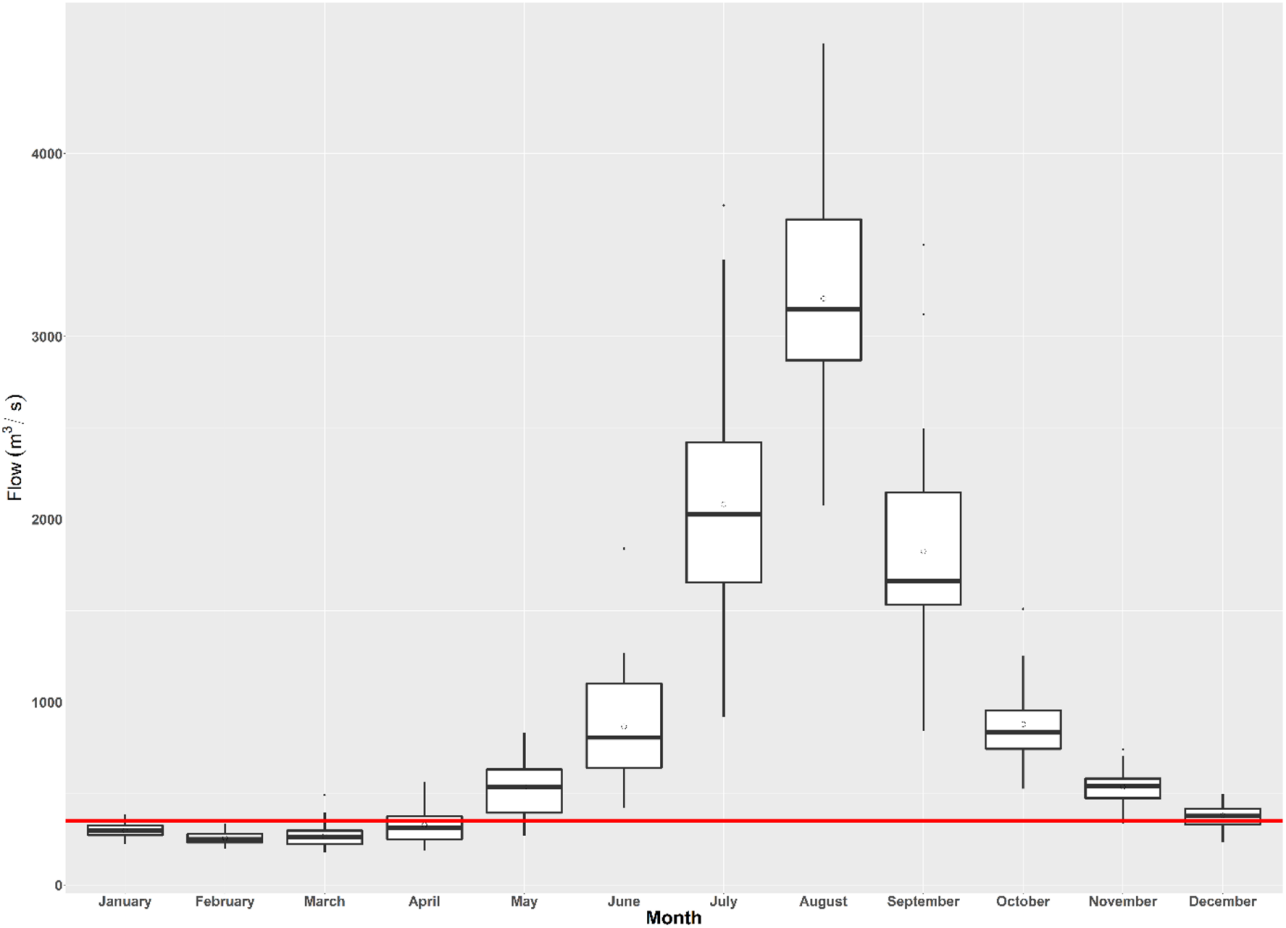
Monthly average 90% time equalled or exceeded flows for the 39 years shows that from January through April, aquatic species suffer from low flows, putting them at risk of evolutionary traps by hugely reducing suitable hydro-physical habitats. The horizontal red line indicates the lower limit of the optimum range.

**Figure 8:**
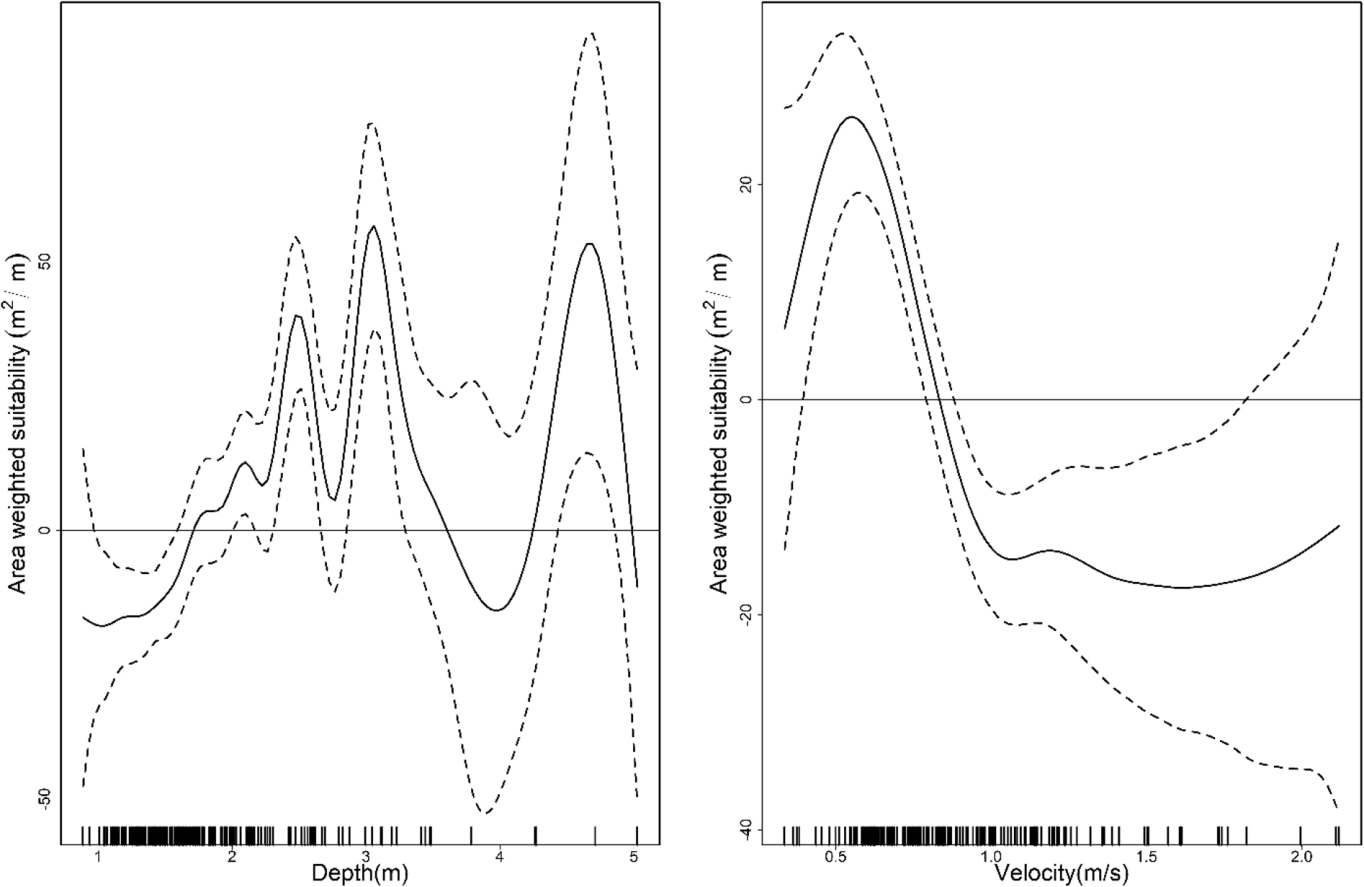
Effects of the depth and velocity on the area weighted suitability (AWS), where several peaks indicate positive and negative (or non-linear relationships) GRD responses.

The GAM models based on AWS velocity and AWS depth explained deviances of 45.7% (R^2^ = 0.502) and 38.1% (R^2^ = 0.458), respectively, with significance of the smooth term (velocity: edf = 6, F = 12.69, *p* < 0.001; depth: edf = 16.33, F = 7.079, *p* < 0.001). Our AWS-flow GAM model explained only 35.6% (R^2^ = 0.431) of the deviance but had a significant predictor smoothing term (flow: edf = 10.04, F = 2.954, *p* = 0.0361). All of the predictors were found to have smoothing terms significantly different from zero (*p* < 0.005) and thus contributed to the model fit of the AWS. All the models exhibit a non-linear relationship, showing positive and negative biological responses to flows, depth, and velocities. Flow < 200 m^3^/s had a negative influence on the AWS. Although flows ranging from 210–230 m^3^/s, 280–350 m^3^/s, and 400–417 m^3^/s contributed positively to the AWS, they might not be proportionally supportive, as they are close to the zero-effect line (Fig. 6). Flows between 351 and 389 m^3^/s had the greatest positive impact on the estimated AWS. Therefore, we recommended flow ranges < 200 m^3^/s, from 210–230 m^3^/s, from 280–350 m^3^/s, and > 400 m^3^/s under a critical flow level of 351–389 m^3^/s as optimum flow levels. Flow ranging from 417–500 m^3^/s also had a negative effect on the estimated AWS. Values higher than 500 m^3^/s likely reflect errors associated with the interpolation of environmental data. Using the 39-year average monthly 90% exceedance flows shows that the winter season (November–May) suffers from low flows (below the optimum flow range, Fig. 7). Therefore, the winter months (January through April), excepting May and November, could be taken as critical low-flow months. Two distinct depth ranges, 1.7–3.7 m and 4.2–5.2 m, had positive effects on the estimated AWS; three depth peaks (2.4 m, 3.2 m, and 4.6 m) contribute the greatest amount to the AWS (Fig. 8). Velocity showed an inverse relation with the estimated AWS. However, velocities up to 0.6 m/s had a positive effect on the estimated AWS and then fell sharply.

## Discussion

Quantifying the adequate amount and timing of water flows to sustain aquatic biota and environmental processes is a global management priority in freshwater ecosystems^12,16^. To cope with the emerging threats to and persistent conservation challenges of freshwater biodiversity, a highly context-dependent, cautious, applied research approach is emphasized^16^. Thus, the process of maintaining ecological integrity is increasingly expensive due to escalating out-of-stream water demand and as a consequence of climate change^26^. To our knowledge, this is the first study to develop flow-ecology responses using top predator or mega-species (body mass > 30 kg) traits to show physiographical and ecologically distinct flow-ecology relationships that support emerging flow-ecology science (e-flow). We present here flow-ecology relationships that are generalizable, quantitative, and able to generate temporally specific predictions of ecological responses to flow alterations. Our approach offers a platform for evaluating the environmental outcomes of water withdrawals or water resource management decisions in larger river systems that share major megafauna, such as river dolphins, particularly rivers in South/East Asia, and South America, which harbour most populations of endangered river dolphins.

We noticed significant changes in the ecological response, as the most fundamental responses to flow variations, across a wide range of temporal and spatial scales, which suggests that our approach is responsive to and can be used to quantify flow-ecology relationships^11^. As habitat acts as a template for species life-history traits or demographic processes^17^, this underpins the importance of the early adoption of flow regulation guidelines for sustaining endangered aquatic life in highly regulated rivers. Thus, our approach, which considers temporally distinctive habitats of the GRD for establishing flow-ecology relationships, could have several advantages for the protection and restoration of the ecological integrity of streams and rivers that share similar hydrological and taxonomic groups (river cetaceans)^27^. These relationships can be applied in two different contexts: to evaluate the probable environmental consequences of flow modification or to establish guidelines for flow restoration in impaired streams. For instance, we used this approach to examine flow release plans [e.g., the proposed minimum flow (10–20% of the monthly minimum flow)] adopted by the massive Upper Karnali hydropower project in our study area. The results indicate that this reference plan is ecologically inadequate and no longer tenable to ensure the long-term functionality of the aquatic ecosystem.

Our findings reveal GRD are more sensitive to depth and velocity of natural flows that cumulatively define the suitable physical habitat template at specific geomorphologies, suggesting a substantial risk to population persistence if the species selects these attributes of flow preferentially under a critical flow. As a result, we observed both positive and negative biological responses to flow alterations, suggesting non-linear flow-ecology relationships, and negative consequences of both high and low flows to the ecology of the GRD (e.g.,^6,28^). This non-linearity likely indicates the salience of time-varying habitat availability for species life-history traits in the environments in which aquatic species occur. We noticed a negative contribution to the AWS from flows beyond certain points, supporting the hypothesis that the natural flow regime does not necessarily optimize all ecological functions of the target species^29^. This offers an opportunity to harvest flow, using caution if the flow exceeds the maximum level. Thus, an approach that specifies when flows are limited and predicts well-defined ecological outcomes using species traits is needed to build trust with managers and gain social support for environmental flows^12^. Using traits from freshwater megafauna species, which offer a preferred multi-species template for fostering demographic processes^17,27^, our approach may allow the construction of reliable flow-ecology relationships that integrate social and ecological demands. However, we stress that the incorporation of non-flow environmental factors, including climatic variability, might further improve the predictive power of our approach^12^.

Significant adverse impacts of water withdrawals during the critical water level season are widely recognized in aquatic species conservation and are the most concerning with larger regulated rivers^30^. This is a significant challenge for aquatic biodiversity or species conservation, since it might cause substantial disproportionate changes in the biological response or result in diminishing ecological returns^11^. We identified ecological thresholds that offer opportunities to address this significant concern in rivers that share similar taxonomic and hydro-physical habitats. Thus, we stress that there is a crucial research and management need globally in the field of aquatic ecology to use species’ life history-specific traits, particularly reproduction and migration traits, to determine the magnitude and timing of appropriate ecological flows^31^.

Strong non-linear ecology-flow relationships across broader temporal scales are often associated with particular requirements of species at various stages of their life histories^11^. Thus, the timing of important life history stages (e.g., the preparatory period for reproduction or the timing of growth or reproduction) should coincide with the optimum water level to sustain aquatic life^16^. The peak reproduction time of the GRD and the timing of the critical water level overlapped to a large extent^32^, which suggests that there is a high risk of mismatch between demographic phenology (i.e., birthing period) and suitable area availability (or usable area), which determines reproductive success. The effects further increase through the synergistic interactions of altered hydrology and anthropogenic impacts, such as overfishing and habitat degradation. Given the importance of water level in determining the breeding and survival success of lotic animals (e.g.,^33^), timely and reasonable water allocation strategies will be needed to sustain aquatic biodiversity while optimizing water availability for human use^34^. To avoid species loss and habitat degradation, conservation approaches that address ecological attributes (e.g., obligatory breeding migrations, use of different habitats at different stages of the life cycle, and the extent of habitat occupancy) are essential^35^. In general, our approach could serve as a fundamental basis in riverine ecosystems, where river dolphin conservation falls short of ecological expectations, while addressing anthropogenic needs. Assuming the temporal patterns of flow variation that determine the habitat templates on which some species adaptations have been established^36,37^, maintaining a pattern of natural variability using proposed ecological thresholds might serve as an effective conservation measure. This approach benefits diverse aquatic taxa by offering temporally dynamic habitats that are useful for the completion of aquatic species life-history events.

We noticed a change in the amount of hydro-physical habitats as the first ecological response to flow variation, which supports the previous study findings^34^. Consequently, we found that GRD is forced to use low-quality habitats over a superior when the flow is reduced, as demonstrated by the considerable low value of use habitat characteristics (depth and velocity) over a habitat with high selection strength (preferred habitats). For example, as the flow declined, GRD were forced to use the habitats with a higher flow velocity as the season progressed towards peak dry season, which had a negative contribution to AWS. The magnitude and direction of such changes are poorly known. In response to this issue, we quantify the variation in ecological responses or usable areas (AWS) as a function of flow fluctuations. Our simulation models predict a loss of 26% of the currently available maximum AWS with a 40% fluctuation in the base flow (536.112 m^3^/s, see detailed flow fluctuation levels in Table 1). To maintain ecological integrity and functionality and retain 80% of the AWS, we recommended maintaining a minimum flow of 383.967 m^3^/s (Table 2) in the Karnali River. To minimize the adverse effects of flow fluctuations, traditionally adopted flow maintenance rules must be revisited in terms of hydro-ecological prospects. Further, while undertaking environmental impact assessments for mega hydropower or water-related projects, investigators must respond to the distribution and abundance of key species, which are affected by the interactions between the species and the changing environment^38^. In our case, the natural variability in flows from January through April seems more critical (below the threshold) for aquatic biodiversity, and perhaps, the severity of environmental impacts can be expected to increase with the duration of low flows during these months. Therefore, hydrological changes need to be considered when assessing the risk to endangered aquatic species in the face of persistent and increasing human activity during the Anthropocene^39^.

As an immediate ecological response by aquatic biota to natural flow alteration (particularly during the dry season when flow naturally reduced and water extraction further accelerated) is the rapid loss of native and sensitive species mediated by the fragmentation of linear habitat corridors that limit their dispersal ability or gene flow between habitats^40,41^. Because of the long-life, low reproductive rate and habitat specialists (highly selective depth and velocity that define their habitats at particular geomorphic), habitat fragmentation certainly exposes river dolphins to the risk of ecological traps, a severe threat to their reproduction and survival. A sharp decline in population size (~ 50%), local extirpation (~ 18% reduction in distribution range), the formation of small isolated groups with a risk of inbreeding, and acute interactions with fisheries were commonly reported consequences of flow reduction in GRD populations in South Asian waterways^32^. Smith^42^ described such ecological traps as “rare hydro-physical” in the same study area of this research, making the GRD more rare. Further, competitive interactions in feeding niches mediated by the reduced flow exacerbate overlap between river dolphins and fisheries, which likely escalates the endangerment and extinction of river dolphins through bycatch or adverse impacts on their health^43^. Although little is known about the flexibility of river dolphins habitat preferences or ability to adjust to changed environmental conditions, the current population size likely determines the fate of these trapped populations in highly regulated river systems^40^. Considering the current rate of decline of South Asian River dolphins (*Platanista gangetica*), the demographic effects of an ecological trap should be substantial and pose an immediate risk of extinction unless social and economic benefits of flow alteration are evaluated against ecological outcomes. Recent extinction of Chinese River dolphins, in addition to the sharp decline of *Platanista gangetica,* suggests immediate attention of conservation or water management authorities in South America, an important home to river dolphins, which is relatively less impacted by water development projects at present. As hundreds of hydroelectric dams have been planned throughout the Amazon, including many in the Orinoco and Tocantins-Araguaia basins^44^, we suggest immediate actions to incorporate flow-ecology relationships in their water use management plans to avoid the risks of native and sensitive aquatic species extinctions.

Considerable interest in flow-ecology analysis exists globally, and our physiographic and ecologically distinctive flow-ecology relationships could reasonably support the global environmental flow guideline development process. Given the complicated non-linear relationships in aquatic ecosystems, relying on the habitat use of megafauna species could offer an opportunity to develop flow-ecology relationships that are scientifically robust, regionally flexible, and ecologically predictable. Foundation species (e.g., top predators), which are structurally and functionally significant taxa, offer critical resources for communities^25^; therefore, ecological thresholds based on the distinctive ecology of river dolphins might serve as a scientific basis for maintaining the environmental integrity of riverine ecosystems. The broader need for concerted, targeted, and timely conservation of freshwater biodiversity has been highlighted globally^45^, and our findings could assist in developing an appropriate and broad biotic integrity plan to improve the resilience of riverine ecosystems. However, ecological responses to flows might differ in different landscapes, so understanding the spatial pattern of flow responses is essential. Our approach can be replicated carefully in other riverine systems that share similar hydrological and geomorphological characteristics, including the presence of mammalian carnivores (e.g., the Indus River dolphin, *Platanista gangetica minor*; Irrawaddy dolphin, *Orcaella brevirostris*; Amazon River dolphin, *Inia geoffrensis*; Tucuxi, *Sotalia fluviatilis*; Araguaian river dolphin, *Inia araguaiaensis* and Bolivian River dolphin, *Inia boliviensis*). As such relationships are unlikely to remain static in a changing environment^46,47^, managers need to anticipate how this dynamism may affect future environmental flow needs and develop appropriate management regimes that are robust to environmental change^34,48^.

## Methods

### Study area

This project was conducted in the downstream segment of the Karnali River basin of Nepal (Fig. 1), which is the largest of Nepal’s three major river systems and is characterized by the steep terrain of the Himalayan Mountains. The highest runoff occurs during the monsoon season (e.g., June–October), and the lowest occurs during the winter season (e.g., December–May). Below the Siwalik Mountain range (a physiographic zone, Fig. 1), a vast network of small tributaries combines to form a single narrow channel of the Karnali River with well-defined banks. Originating from the Tibetan Plateau, the Karnali River is the largest tributary to the Ganges River in India, which harbours the most significant density of GRDs in the world. The lower Karnali River basin provides the furthest upstream range for GRDs, critically endangered gharials (*Gavialis gangeticus*), smooth Indian otters (*Lutrogale perspicillata*), and 36 native fish species^49^. The GRD population size in the Karnali River has declined from 26 to six individuals^50^. Such a sharp decline in the GRD population is due to the effects of habitat degradation, mainly from water-based development projects (i.e., water diversion,^51^). Concurrently, several upstream development projects are proposed, under construction, or completed [e.g., planned: the Karnali Chisapani multipurpose dam, 10,800 megawatt (MW); under construction: the upper Karnali hydropower project, 900 MW, and Bhari Babai diversion project; completed: Rani Jamara Kulariya irrigation intakes] and further threaten downstream aquatic life. All projects adopt traditional preconstruction environmental impact assessments procedure to define flow proportions (generally 10–20% of natural regimes) anecdotally and unscientifically. Thus, traditional flow proportions might be inadequate to sustain native aquatic biodiversity. Our study focused on the lower catchment area of the Karnali River basin, which is downstream from all megaprojects. All measurement protocols, including dolphin observation methods, were carried out in accordance with the Department of National Parks and Wildlife Conservation, Government of Nepal, guidelines and regulations. Habitat measurement protocols, including dolphin observation methods, were approved by the Department of National Parks and Wildlife Conservation, Government of Nepal (No 1129; 12 December 2016).

### Available habitat assessment

Reduced water levels during the low-water season (e.g., December–May) escalate threats to aquatic biota by limiting physical habitat availability. Here, habitat refers to the hydro-physical habitat, which is defined by the flow and depth interactions at a particular geomorphic condition over space and time. Therefore, habitat availability (i.e. the area accessible to species) is assumed to be the greatest bottleneck, critically limiting species reproduction and survival^42,51^. We measured the available habitats in the low-water season when suitable habitat is critically limited (i.e., December–May in 2018/2019), excluding the monsoon season (June–November). Further, to capture dynamic flow variation within the dry (i.e., low) water season, we selected three temporal periods—March (mid dry season), May (late dry season), and December (early dry season)—based on 39 years of flow records available from the Department of Hydrology, Government of Nepal. Assessing available habitat includes habitat mapping and bank and instream surveys. We divided the study area into three segments [upper segment (S1): length = 11 km, average width = 218 m; middle segment (S2): length = 29 km, average width = 121 m; and lower segment (S3): length = 10 km, average width = 198 m; Fig. 1] based on uniform flow and channel geomorphology mapped along the selected stretch of the river. The three segments vary hydrologically and structurally. S1 consists of river channels with natural flows without any infrastructural diversion. Because of water diversion operations (e.g., Rani Jamuna irrigation intake and several traditional agricultural irrigation channels) and distributaries, the natural flow volume in S2 was low compared to that in S1. S3 benefited slightly from distributaries and received more water than S2.

Within each segment, the study reach (the linear segment where cross-sections are established) was established in such a way that the length of each reach was at least higher than the mean width (so the number varies among segments) of the respective segment. We also tried to maintain relatively similar flow at the top and bottom of the reach. Within each reach, random cross-sections were established to capture the hydraulic properties based on flow variation. As the flow variability of the stream increased, the number of cross-sections increased, and each section was kept at least 300 m apart from the other sections. Therefore, the number of cross-sections was based on the flow variation within a reach instead of the length of the reach. Bank and instream surveys started in an upstream direction, wherein directional readings of the cross-sections were noted. For the bank and instream measurements, pin heights were established at either side of the cross-section using GPS and a permanent reference marker for repeated flow measurements. Water surface elevations were estimated using a total station (an optical instrument for land surveying; Leica 772737 Builder 503) across the pin heights for each cross-section required for hydrological simulation. A new benchmark was established for each effort to measure the water surface elevation at each cross-section. The total number of cross-sections examined for the available habitats was 177 (March = 60, May = 47, and December = 60). The hydraulic parameters (see habitat characterization section below) at each cross-section were measured using a RiverSurveyor S5 acoustic water current profile reader [Sontek, Acoustic Doppler Profiler (ADP S5)], which records hydro-parameters continuously at a cell size between 0.02 to 0.5 m offering complete underwater available hydro-physical profile.

### Occupied habitat assessment

We conducted a GRD population survey to capture occupied (selectivity) habitat characteristics (n = 97) at three temporal scales (previously mentioned) using the developed approach^24^. Within each temporal scale, we conducted three replications to capture the temporal and spatial variability in the characteristics of the occupied habitat. When we first detected dolphins, we observed surfacing behaviours for at least five minutes before establishing a cross-section. The habitats that were used for at least five minutes were considered occupied habitats, and then cross-sections were established to measure habitat characteristics using the ADP. If the dolphins disappeared after the location of the first sighting in less than five minutes, we excluded those habitats from our analysis. The Dolphin observation (only observation done) protocols were approved and permitted by the Department of National Parks and Wildlife Conservation, Government of Nepal.

### Data analysis

#### Data preparation and software

The ADP S5 hydraulic data were imported into *Excel* databases (Microsoft v. 2010) to format for *System for Environmental Flow Analysis* (SEFA, version 1.5; Aquatic Habitat Analysts Inc.) software. All the hydraulic properties [depth (m), velocity (m/s), wetted perimeter-WP (m), width (m), cross-sectional area-CSA (m^2^), Froude number, and discharge (m^3^/s)], suitability, and flow regime determination were calculated using SEFA software and analysed at the cross-section and segment levels. The average flow of each segment was used as a base flow while running the habitat simulation model for the respective segment. We found critical flows (< 210 m^3^/s, an insufficient flow that has a negative contribution to habitat suitability) in December and March and excess flows (> 417 m^3^/s, excess flow with a negative contribution to habitat suitability) in May. Therefore, the habitat retention hydraulic simulation model was performed only with excess flow (for May) using 39 years of 90% exceedance flow (the flow that is equaled or exceeded 90% of the time).

#### Habitat characterization

The cross-sectional hydro-physical parameters—width, flow, depth, velocity, wetted perimeter, cross-sectional area, and habitat (types)—were reported spatially and temporally. The habitat type (e.g., pool, run, and riffle) was classified based on the Froude number (F_r_), where Froude is an index of hydraulic turbulence (the ratio of velocity by the acceleration of gravity). Points with Froude numbers exceeding 0.41 were considered riffles, points with Froude numbers less than 0.18 were considered pools, and intermediate values were classified as run habitats. The proportion of run, riffle, and pool habitats within each study reach was calculated from the Froude numbers. The GRD’s seasonal hydro-physical habitats were characterized using basic descriptive statistics (mean and 95% CI). The variation in these hydraulic parameters among seasons, habitat types, and segments was examined by an analysis of variance (ANOVA), and post hoc pairwise comparisons were performed using Tukey’s honestly significant difference (HSD) test. A two-way ANOVA test was used to investigate any interactive effects of season and habitat on hydraulic variations. The level of significance was set at *p* < 0.05 for all the statistical tests. All the analyses were conducted using R Studio.

#### Hydro-physical habitat modelling

The GRD’s suitable habitat (i.e., habitat selectivity) is defined as the range of hydro-physical conditions in which GRDs are most likely to be found (excluding water quality). The habitat simulation approach comprised two steps: developing the habitat suitability curve (HSC) and estimating the area-weighted suitability (AWS) using the HSC and flow relationships. The HSC was developed using the GRD *occupied* and *available* habitat datasets. To develop the HSCs, understanding the strength of selection for a particular habitat is essential. Therefore, we measured habitat selectivity (*w*) at equal intervals of both depth and velocity to measure the preference strength (preferred category) for a specific category of habitat. Habitat selectivity was calculated as the proportion of a habitat class that was occupied divided by the proportion of that category available in the whole sample^52^. A value of *w* = one indicates neutral preference, *w* < one indicates that the habitat was used less commonly than expected by chance, and habitats with *w* > one are used more frequently than expected by chance. Using the selectivity values (*w*), we transformed the depth and velocity categories into a binary scale of zero and one. We assigned a value of one to those categories for which *w* > one and zero to those categories for which *w* ≤ one. By assigning one and zero to each group, we developed an HSC to calculate the area weighted suitability (AWS) at each measured point. Hydraulic habitat suitability is expressed as AWS in terms of usable area in metres of width or square metres per metre of reach (m^2^/m).

To obtain the AWS value for the reach, we multiplied the combined suitability index (CSI, which is the product of the suitability of depth and velocity at a point) and the proportion of the reach area represented by that point. Using a 39-year average base flow of 536.11 m^3^/s (90% exceedance flow) in May, we predicted the fluctuation (decrease by 10%) in the currently available maximum AWS (i.e., 22.718 m^2^/m, AWS of May) in the range of flows from 200–900 m^3^/s. We simulated the AWS in this particular range because this range represents the 39-year low and maximum values of the 90% exceedance flow for the low-water season (November–May). Covering this variation over a broader scale increases the applicability of our ecological thresholds across time. Using the same base flow and range, we also estimated the minimum flows that retain various standards (%) of habitat protection. Further, we also determined the minimum flow that provides the maximum AWS for the low-water season.

#### Ecological thresholds using flow-ecology relationships

As water depth and velocity are the result of instream habitat features, such as pools, riffles, and runs, we only incorporated depth and velocity when estimating the hydraulic habitat suitability. Additionally, GRD habitat selection is strongly guided by the depth and velocity of a river section^24,51^. Generalized linear models (GLMs) using logit functions were used to examine the relationship between GRD presence and hydraulic properties (depth and velocity). Four different GLMs (depth, velocity, depth*velocity, and depth + velocity) were developed, and the Akaike information criterion (AIC) was used to select the best models. The additive effect of depth and velocity on the GRD presence was found in the model with the best performance; therefore, we further used a generalized additive model (GAM) to capture the possible non-linear influence of depth and velocity on GRD presence. Because of the possibility of both linear and non-linear relationships^11^, we again used a GAM to capture the functional relationships between ecology (AWS) and flow. The degree of smoothness for all the GAMs identified by the iterative approach (up to 25 smoothing factors were checked) and the selected smoothing parameter (i.e., 20 for all the GAM models) that yielded a significant covariate (at the 0.005 level of significance) explained the maximum deviance and adjusted R^2^. Both the GLM and GAM models were fitted using the *lm* and *mgcv* packages in R Studio.

## Data Availability

All data supporting the conclusions of this article are within the paper.
